# An audit of sample sizes for pilot and feasibility trials being undertaken in the United Kingdom registered in the United Kingdom Clinical Research Network database

**DOI:** 10.1186/1471-2288-13-104

**Published:** 2013-08-20

**Authors:** Sophie AM Billingham, Amy L Whitehead, Steven A Julious

**Affiliations:** 1 School of Medicine, The University of Sheffield, Beech Hill Road, Sheffield S10 2RX, UK; 2 Medical Statistics Group, School of Health and Related Research (ScHARR), University of Sheffield, Regent Court, Regent Street, Sheffield S1 4DA, UK

**Keywords:** Pilot, Feasibility, Sample size, UK

## Abstract

**Background:**

There is little published guidance as to the sample size required for a pilot or feasibility trial despite the fact that a sample size justification is a key element in the design of a trial. A sample size justification should give the minimum number of participants needed in order to meet the objectives of the trial. This paper seeks to describe the target sample sizes set for pilot and feasibility randomised controlled trials, currently running within the United Kingdom.

**Methods:**

Data were gathered from the United Kingdom Clinical Research Network (UKCRN) database using the search terms ‘pilot’ and ‘feasibility’. From this search 513 studies were assessed for eligibility of which 79 met the inclusion criteria. Where the data summary on the UKCRN Database was incomplete, data were also gathered from: the International Standardised Randomised Controlled Trial Number (ISRCTN) register; the clinicaltrials.gov website and the website of the funders. For 62 of the trials, it was necessary to contact members of the research team by email to ensure completeness.

**Results:**

Of the 79 trials analysed, 50 (63.3%) were labelled as pilot trials, 25 (31.6%) feasibility and 14 were described as both pilot and feasibility trials. The majority had two arms (n = 68, 86.1%) and the two most common endpoints were continuous (n = 45, 57.0%) and dichotomous (n = 31, 39.2%). Pilot trials were found to have a smaller sample size per arm (median = 30, range = 8 to 114 participants) than feasibility trials (median = 36, range = 10 to 300 participants). By type of endpoint, across feasibility and pilot trials, the median sample size per arm was 36 (range = 10 to 300 participants) for trials with a dichotomous endpoint and 30 (range = 8 to 114 participants) for trials with a continuous endpoint. Publicly funded pilot trials appear to be larger than industry funded pilot trials: median sample sizes of 33 (range = 15 to 114 participants) and 25 (range = 8 to 100 participants) respectively.

**Conclusion:**

All studies should have a sample size justification. Not all studies however need to have a sample size calculation. For pilot and feasibility trials, while a sample size justification is important, a formal sample size calculation may not be appropriate. The results in this paper describe the observed sample sizes in feasibility and pilot randomised controlled trials on the UKCRN Database.

## Background

The National Institute of Health Research Evaluation, Trials and Studies Coordinating Centre (NETSCC) defines a pilot trial for a randomised controlled trial (RCT) as *‘a version of the main study…run in miniature to test whether the components of the study can all work together’* and a feasibility study for an RCT as *‘research done before a main study to answer the question “Can this study be done?”.*[1] However, whilst some authors, including Arain et al. [2] recommend these definitions, in truth there is no consensus. Stallard [3] reports a reason for this as being in part, due to the wide variety of purposes for which pilot trials are undertaken.

Thabane et al. [4] give a number of reasons as to why pilot trials may be conducted. They state that conducting a pilot trial before a main study can increase the likelihood that the main study will be a success, and may potentially help to avoid ‘doomed’ main trials. They also state that in many cases, pilot trials are performed in order to generate data for sample size calculations in the main study.

Prescott and Soeken [5] meanwhile, suggest five pilot trial aims based on a review of then-current nursing research text books including: a feasibility assessment; adequacy of instrumentation and answering methodological questions.

To address the aims of a pilot trial a sample size justification is required. Hertzog [6] highlights that there is little published guidance on for a pilot trial sample size. However, when applying for funding for a pilot trial, a review panels would expect a justification for the planned sample size. This justification could be based on a number of methods:

• Hertzog [6] recommends the Julious and Patterson [7] method of using confidence intervals for a given precision constructed around the anticipated value to set the sample size;

• Stallard [3] proposes that the sample size should be approximately 0.03 times that the sample size planned to be included in the definitive study;

• Browne [8] gives a general rule is to take a minimum of 30 patients to estimate a parameter;

• Julious [9] recommends a minimum sample size of 12 per group as a rule of thumb and justifies this based on rationale about feasibility and precision about the mean and variance;

• Sim and Lewis [10] suggest a sample size of at least 50 per group.

Setting an appropriate sample size for any study is important. If a study is too large it may be judged to be unethical as participants may be unnecessarily exposed to risks and burdens [11]. There is the additional issue that setting the sample size too high may lead to a preventable failure to reach the recruitment target [12]. While Julious [9] highlights that a sample size that is too small will have an imprecisely estimated variance, which could impact on the design of a future definitive study.

This paper aims to build on the work of Lancaster et al. [12] who reviewed pilot trials published from 2000 to 2001 in seven major journals and Arain et al. [2] who revisited the same seven journals from 2007 to 2008 to see if there had been any change in how pilot trials were reported.

Arain et al. [2] concluded that pilot trials are poorly reported and that the authors are often not explicit as to the purpose of their pilot trial. They also found that sample size calculations were only performed and reported in 35% of the trials and that those identified using the key word ‘pilot’ were more likely to have a pre-study sample size calculation.

Using data from the United Kingdom Clinical Research Network (UKCRN) Database we extend the work of Lancaster et al. [12] and Arain et al. [2] by investigating the sample size of pilot and feasibility trials for RCTs currently running in the United Kingdom (UK). The aim was to investigate on-going sample sizes for pilot/ feasibility trials in the UK. Although as discussed, there are definitions of pilot and feasibility available, we recognise that in reality the terms are often used interchangeably. However, Arain et al. [2] found that there were some differences between the designs of studies labelled pilot and feasibility. Therefore, in this investigation we will distinguish between pilot and feasibility trials in the analysis. We will further look at whether the sample sizes chosen varies between the two study types (pilot or feasibility), as defined by the principal investigator in their UKCRN Database entry.

The paper will also investigate if the sample size chosen for the trial is influenced by factors such as how the trial is funded or the type of endpoint.

The three research aims of the paper are:

1 To describe the sample sizes set for trials labelled pilot versus feasibility

2 To describe the sample sizes set for trials with a dichotomous compared to a continuous endpoint

3 To describe the sample sizes set in trials funded by industry, public bodies or charities.

## Methods

### Trial identification

The UKCRN database, [http://public.ukcrn.org.uk/search/ (data last accessed, 20 March 2013)] [13] was used to identify pilot and feasibility trials currently ongoing in the UK. The database comprises of the National Institute for Health Research (NIHR) portfolio in England, and the corresponding portfolios of Northern Ireland, Scotland and Wales. The studies benefit from the support given by the clinical research network (CRN), however, it is not compulsory for researchers to register with the UKCRN [14]. The database is accessible by anyone online through the URL listed above. The search was conducted on the 17th May 2012 using the key words ‘Pilot’ or ‘Feasibility’ in the title or research summary. These were the same key words used by Lancaster et al. [12] and Arain et al. [2] and were used here to maintain consistency with previous research.

The search results were exported to Excel and the studies were sorted first by primary study design in order to separate the interventional trials from the observational studies. They were then sorted by active status: in order to separate the open from the closed trials.

The open interventional trials were then assessed against the eligibility criteria as set out below. After the trials had been assessed against the inclusion criteria the eligible trials were exported into SPSS version 18.0 [15] for analysis.

Trials were eligible for further analysis if:

• They were randomised controlled trials;

• They were currently recruiting participants;

• They were classified as interventional;

• The participants were not healthy volunteers;

• They were not cluster randomised trials.

Trials were only included in the analysis if they were open in order to get the most up to date picture of sample sizes being used for pilot trials in the UK. Trials being conducted on healthy volunteers were not included as these are not usually efficacy studies. Cluster randomised trials were excluded from further analysis as they tend to require much larger target sample sizes (in terms of numbers of patients not clusters) than those trials which randomise patients individually. Cluster randomised trials also have different methodological issues and concerns when undertaking a pilot trial – for example to estimate the intra-class correlation (ICC).

### Data extraction

Data on the target sample size and components of the trials that might influence the target sample size such as, type of end point, funder, number of treatment arms and disease area were collected.

The information was extracted from the research summary of the UKCRN database when available. Forty-four of the trials provided an International Standard Randomised Controlled Trial Number [ISRCTN, http://isrctn.org/ (Date last accessed 23rd March 2013)] these were then used to conduct individual searches of the ISRCTN Register, when information was missing.

To complement the search of the UKCRN database, an Internet search was undertaken to find the trial or other websites when information about the trial was missing from the UKCRN. Additional websites used included the US clinicaltrials.gov and the website of the funder of the study.

After conducting all of these searches 62 (75%) of the trials did not have complete information and so, in these cases, the principal investigator or funder(s) were contacted by email for the study protocol in question, in all cases responses were received.

### Analysis plan

Medians and ranges were calculated overall for the different types of trial and then broken down by endpoint and whether the trial was public or industry funded.

## Results

**Figure 1:**
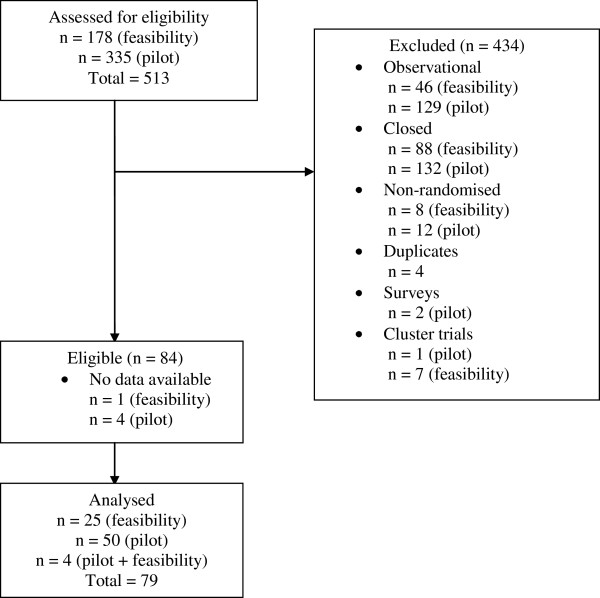
Flow diagram showing the flow of trials through the review.

The search of the UKCRN database yielded 178 studies with the search term ‘feasibility’ and 335 studies with the search term ‘pilot’. After eliminating duplicates, removing any studies not meeting the inclusion criteria and studies where no data were available, 83 trials went on to be analysed. Studies with no data available, means that although the trial was registered, no information regarding the trial was listed or available from other sources. In these cases (n = 5) the trial investigators were contacted however, none of these replied and the trials were assessed as ineligible. Of those eligible, 26 had been labelled as a feasibility by the investigators, 53 had been labelled a pilot trial and 4 had received the label of both a pilot and a feasibility. Figure 1 shows the flow of trials through the review.

### Trial characteristics

Table 1 summarises the characteristics of the trials that met the inclusion criteria. The majority of the trials (n = 68, 86.1%) consisted of two arms: one experimental treatment and one control treatment, whether that control be active, a placebo or usual care. The majority of the trials had either a continuous endpoint (n = 45, 57.0%) or a dichotomous endpoint (n = 31, 39.2%).

**Table 1:** Trial characteristics of the studies included in the final analysis

		**Description of preliminary study**							
			**Pilot**		**Feasibility**		**Both**		**Total**
		**n**	**(%)**	**n**	**(%)**	**n**	**(%)**	**n**	**(%)**
Number of arms	Two	39	78.0	25	100.0	4	100.0	68	86.1
	Three	10	20.0	0	0.0	0	0.0	10	12.7
	Four	1	2.0	0	0.0	0	0.0	1	1.3
Type of trial	Health technology	34	68.0	23	92.0	3	75.0	60	75.9
	Drug	16	32.0	2	8.0	1	25.0	19	24.1
Disease area	Stroke	4	8.0	1	4.0	0	0.0	5	6.3
	Mental health	11	22.0	6	24.0	1	25.0	18	22.8
	Oncology	4	8.0	4	16.0	0	0.0	8	10.1
	Respiratory	3	6.0	1	4.0	0	0.0	4	5.1
	Oral & Gastrointestinal	3	6.0	2	8.0	0	0.0	5	6.3
	Dementias	3	6.0	1	4.0	0	0.0	4	5.1
	Cardiovascular	2	4.0	2	8.0	1	25.0	5	6.3
	Primary care	5	10.0	2	8.0	0	0.0	7	8.9
	Musculoskeletal	4	8.0	1	4.0	0	0.0	5	6.3
	Other	11	22.0	5	20.0	2	50.0	18	22.8
Type of end point	Dichotomous	15	30.0	12	48.0	4	100.0	31	39.2
	Continuous	35	70.0	10	40.0	0	0.0	45	57.0
	Time-to-event	0	0.0	1	4.0	0	0.0	1	1.3
	Other	0	0.0	2	8.0	0	0.0	2	2.5
Funder	Industry	11	22.0	1	4.0	1	25.0	13	16.5
	Public	27	54.0	17	68.0	3	75.0	47	59.5
	Charity	12	24.0	7	28.0	0	0.0	19	24.1

The most common disease areas for the trials were, mental health (n = 18, 22.8%) oncology (n = 8, 10.1%) and primary care (n = 7, 8.9%). Although there was a large variety of clinical areas being investigated as shown in Table 1. Approximately 75% of the trials were health technology trials (n = 60) with drug trials making up the remaining percentage (n = 19).

Most of the trials (n = 47, 59.5%) were publicly funded, with the remaining trials being funded by either a charity (n = 19, 24.1%) or industry (n = 13, 16.5%).

### Sample size

The UKCRN database provided a target sample size for each trial in their research summary. However, there were no data available to explain why each target sample size had been chosen.

In approximately 11% of cases (n = 9), the researchers had recruited more patients to date than they initially said would be required. These trials ranged from having a sample size per arm of 15 to 100.

Data were first gathered on the target sample size per arm for pilot and feasibility trials. Those trials labelled pilot were found to have a smaller sample size per arm (median of 30; range 8 to 114 participants) than those labelled feasibility (median of 36; range 10 to 300 participants), these results and the inter-quartile ranges (IQR) are shown in Table 2. Over all, the median sample size per arm was found to be 30 (range 8 to 300).

**Table 2:** Median sample size per arm according to type of study, funder and endpoint

			**Sample size per arm**	
		**n**	**Median**	**(IQR) [Range]**
Trial description	Pilot	50	30	(20, 45) [8, 114]
	Feasibility	25	36	(25, 50) [10, 300]
	Both	4	49	(36, 61) [23, 72]
Type of endpoint	Dichotomous	31	36	(25, 50) [10, 300]
	Continuous	45	30	(20, 50) [8, 114]
Funder	Industry	13	30	(16, 31) [8, 100]
	Public	47	36	(25, 60) [10, 300]
	Charity	19	30	(20, 45) [15, 52]

Data on the median sample size were then analysed according to funder. The results are shown in Table 2. Publicly funded pilot trials have a median sample size of 36 (range 10 to 300 participants) and industry funded pilot trials have a median sample size of 30 (range 8 to 100 participants).

The data were also analysed with regard to type of endpoint used. The results are shown in Table 2. Those studies with a dichotomous endpoint had a median sample size larger than those with a continuous endpoint.

Finally, the data were broken down by both funder and endpoint. The results are shown in Table 3. Public pilot trials with a continuous endpoint were on average larger than industry funded pilot trials with a continuous endpoint (medians of 30 and 23 respectively). The same applies to the public and industry funded pilot trials with a dichotomous endpoint (medians of 36 and 25 respectively). Feasibility trials with a dichotomous endpoint in publicly funded trials are on average larger than the equivalent continuous endpoint trials.

**Table 3:** Median sample sizes per arm of pilot and feasibility studies by endpoint and funder

				**Sample size per arm**	
			**n**	**Median**	**(IQR) [Range]**
Pilot	Industry	Dichotomous	5	25	(25, 30) [10, 90]
		Continuous	6	23	(15, 31) [8, 100]
	Public	Dichotomous	6	36	(30, 42) [20, 60]
		Continuous	21	30	(20, 60) [15, 114]
Feasibility	Industry	Dichotomous	0	.	.
		Continuous	1	30	.
	Public	Dichotomous	9	50	(30, 70) [25, 300]
		Continuous	6	43	(15, 60) [10, 60]

## Discussion

Building on the work of Lancaster et al. [12] and Arain et al. [2] the trials analysed in this paper were trials currently running in the United Kingdom on the date the search was conducted, giving us a wide range of information regarding target sample sizes. All the trials that met the inclusion criteria stated a target sample size for their trial within their research summary. Although it is not a requirement in none of the summaries was there a justification given for the target sample size given.

Moore et al. [16] highlighted that it is not unusual for study proposal reviewers to come across a statement such as “No sample size justification is needed because of the pilot nature of the proposed study”, but they state that pilot trials are not exempt from needing a clear rationale for the number of patients to be included. However, Arain et al. [2] discovered that only a small proportion of published pilot trials report pre-study sample size calculations as most journal editors state that it is not mandatory criterion for publication.

An investigation of the expected benefits, risks and costs of the study is required to justify a target sample size [16]. However, it is important to remember that a target sample size for a pilot or feasibility study is only a preliminary figure and has a great degree of uncertainty. For example, the researchers may find that more participants drop out than first presumed. We have shown that target sample sizes vary for preliminary trials. Considering the median sample sizes for pilot and feasibility trials our data shows that on average feasibility studies are larger than pilot trials: although there is wide variability in the sample sizes across all types of trial. The median sample size per arm across all the types of study was 30.

With regards to target sample size according to funder, a study of registered drug trials by Bourgeois et al. [17], across a wide variety of types of trial, found that those funded by industry were more likely to have a larger sample size than those funded by government sources. However, our analysis indicated that publicly funded pilot trials were larger than industry funded pilot trials.

Campbell et al. [18] describe sample size calculations for studies that have dichotomous, ordered categorical and continuous endpoints. They state that approximately 30% fewer patients are required for a study with a continuous endpoint – in our research we found that for a dichotomous endpoint compared to a continuous the median sample size was 20% bigger.

Looking at the differences in sample size according to type of primary endpoint and funder we found that there is a larger difference in sample size between trials with a dichotomous endpoint compared to a continuous endpoint for publicly funded trials compared to industry funded trials.

It would be beneficial to follow-up the pilot and feasibility trials discussed in this paper to see how many go on to be published – to see if there is a difference between those published and not published. Another possible extension would be to investigate the different sample sizes of trials dependent on whether the primary endpoint of the trial is based on efficacy or feasibility.

The limitations of this study include the fact that only one trial registry was used to collect the data meaning that it is possible that eligible trials that were not registered with the UKCRN are missing from the analysis. If these trials differ in some way from the trials listed on the UKCRN then this could affect the conclusions made. The database used only trials being carried out in the UK, which could also affect the generalisability of the results. The search was only carried out by one reviewer and was not repeated to check for accuracy. In addition, only two search terms were used; pilot and feasibility therefore, some trials labelled for example, exploratory or preliminary could have been missed during data extraction. However, these search terms were used to maintain consistency with previous research [2,12].

## Conclusion

All trials should have a sample size justification. Not all trials however need to have a sample size calculation. For feasibility and pilot trials, while a sample size justification is important, a formal calculation may not be appropriate. In our study we found that the median pilot study sample sizes for two arm trials were 36 and 30 per arm respectfully for dichotomous and continuous endpoints.

## Competing interests

The authors declare that they have no competing interests.

## Authors’ contributions

SB identified the trials, extracted the data and performed the analyses. AW created Tables 2, 3 and helped to draft the manuscript. SJ helped to draft the manuscript. All authors read and approved the final manuscript.

## Pre-publication history

The pre-publication history for this paper can be accessed here:

http://www.biomedcentral.com/1471-2288/13/104/prepub
